# Establishment of a two-dimensional PCR method for simultaneous detection of nine sexually transmitted disease pathogens: insights into coinfection rates and epidemiological trends in HPV screening

**DOI:** 10.1128/spectrum.00237-25

**Published:** 2025-04-24

**Authors:** Shuang Yao, Jun Zhang, Lili Pan, Yang Yu, Guanghua Luo

**Affiliations:** 1 Clinical Medical Research Center, The Third Affiliated Hospital of Soochow University, Changzhou, China; National Chung Hsing University, Taichung, Taiwan; Azeezia Medical College Hospital, Kollam, Kerala, India; Karuna Medical College, Palakkad, Kerala, India

**Keywords:** 2D-PCR, sexually transmitted diseases, HPV, co-infection, epidemiology

## Abstract

This study developed a two-dimensional PCR (2D-PCR) method for simultaneous detection of nine sexually transmitted disease pathogens (STDPs) in cervical brush samples collected after human papillomavirus (HPV) screening, aiming to evaluate co-infection rates and epidemiological trends. The 2D-PCR assay was optimized under single-tube closed conditions, with sensitivity and accuracy validated. The prevalent nine STDPs were assessed in 2,193 females undergoing routine gynecological inspections and HPV screening. Statistical analysis revealed correlations between HPV genotypes and identified pathogens. Results showed detection limits (LODs) of 10²–10³ copies/μL for STDPs, with high concordance to triplex real-time PCR (Kappa = 0.90). Overall, 48.6% of samples tested positive for ≥1 pathogen, with 36.02% positive for ≥1 STDP. The most prevalent STDPs were *Ureaplasma parvum*/*Ureaplasma urealyticum* (27.04%), *Mycoplasma hominis* (3.42%), and *Trichomonas vaginalis* (0.23%). HPV-positive individuals exhibited higher STDP infection rates (46.32%) than HPV-negative counterparts (32.86%). Significant associations were observed between HPV infection and *U. parvum/U. urealyticum* or *M. hominis*. HPV 52 was the predominant genotype in STDP-infected individuals (6.2%), with genotypes 52, 53, 6, 11, 42, 43, and 61 significantly more prevalent in STDP-positive groups. The 2D-PCR method proved sensitive and specific for multiplex STDPs detection, while epidemiological data highlighted significant STDP-HPV correlations.

**IMPORTANCE:**

This study introduces the first single-tube 2D-PCR method for efficient, high-throughput detection of nine STDPs, addressing a critical gap in co-infection diagnostics. The high prevalence of STDPs and their strong association with human papillomavirus (HPV) infection underscore the clinical relevance of co-pathogen screening, particularly in HPV-positive populations. The significant linkage between *U. parvum*/*U. urealyticum*, *M. hominis*, and HPV infection suggests potential synergistic mechanisms influencing HPV persistence or progression. Notably, the predominance of HPV 52 and increased prevalence of genotypes 53, 6, 11, 42, 43, and 61 in STDP-infected individuals highlight how pathogen co-infections may modulate HPV genotype distribution. These findings provide a robust tool for integrated STDPs/HPV screening and offer epidemiological insights to guide targeted prevention strategies, ultimately enhancing management of sexually transmitted infections and HPV-related cervical disease.

## INTRODUCTION

Sexually transmitted diseases (STDs) have a profound effect on reproductive and sexual health worldwide. New data on STDs from WHO show 374 million new cases per year, including 128 million cases of chlamydia, 82 million cases of gonorrhea, 156 million cases of trichomoniasis, and 7 million cases of syphilis (1). Sexually transmitted infections (STIs) continue to be a major public health burden in terms of mortality, morbidity, and quality of life, especially in developing countries.

The overall infection rate of high-risk human papillomavirus (HPV) in mainland Chinese women was 19% (2). With the global promotion and commercialization of the HPV vaccine, the importance of screening for HPV is increasingly recognized. Studies suggest that high-risk HPV is associated with the progression of cervical cancer, but it is not the only factor in the development of this disease (3). Chronic infections from other STIs, which lead to an inflammatory microenvironment, also contribute to the progression of cervical cancer and other reproductive system diseases (4). Therefore, this study has established an economical and simple detection method that repurposes “waste” cervical brush samples obtained after HPV screening. This method is capable of simultaneously testing nine types of STDPs from extracted DNA samples, including *Ureaplasma parvum*/*Ureaplasma urealyticum, Mycoplasma hominis, Trichomonas vaginalis, Mycoplasma genitalium*, *Neisseria gonorrhoeae, Chlamydia trachomatis, Herpes simplex virus type I* (HSV-1), and *Herpes simplex virus type II* (HSV-2). Patients or individuals undergoing health check-ups only require one sampling procedure to achieve concurrent detection of HPV and STDPs, reducing patient discomfort and significantly cutting down on economic costs. The detection method, based on the principles of 2D-PCR, enables single-tube simultaneous testing of nine STDPs. It is characterized by its simplicity, short reaction time, low cost, independence from additional product identification instruments, and avoidance of complex analysis procedures, while ensuring reliable and stable results.

Utilizing this method, this study conducted STDPs detection on population samples from the Changzhou region of Jiangsu Province, China, who underwent HPV screening between 2022 and 2023. The aim was to assess the co-infection rate of STDPs and HPV in this region and to analyze the epidemiological trends of STD in the population. These data provide a scientific basis for the development of effective prevention and treatment strategies for HPV and other STIs.

## RESULTS

### Establishment of 2D-PCR method for single-tube detection of nine STDPs

**Fig 1:**
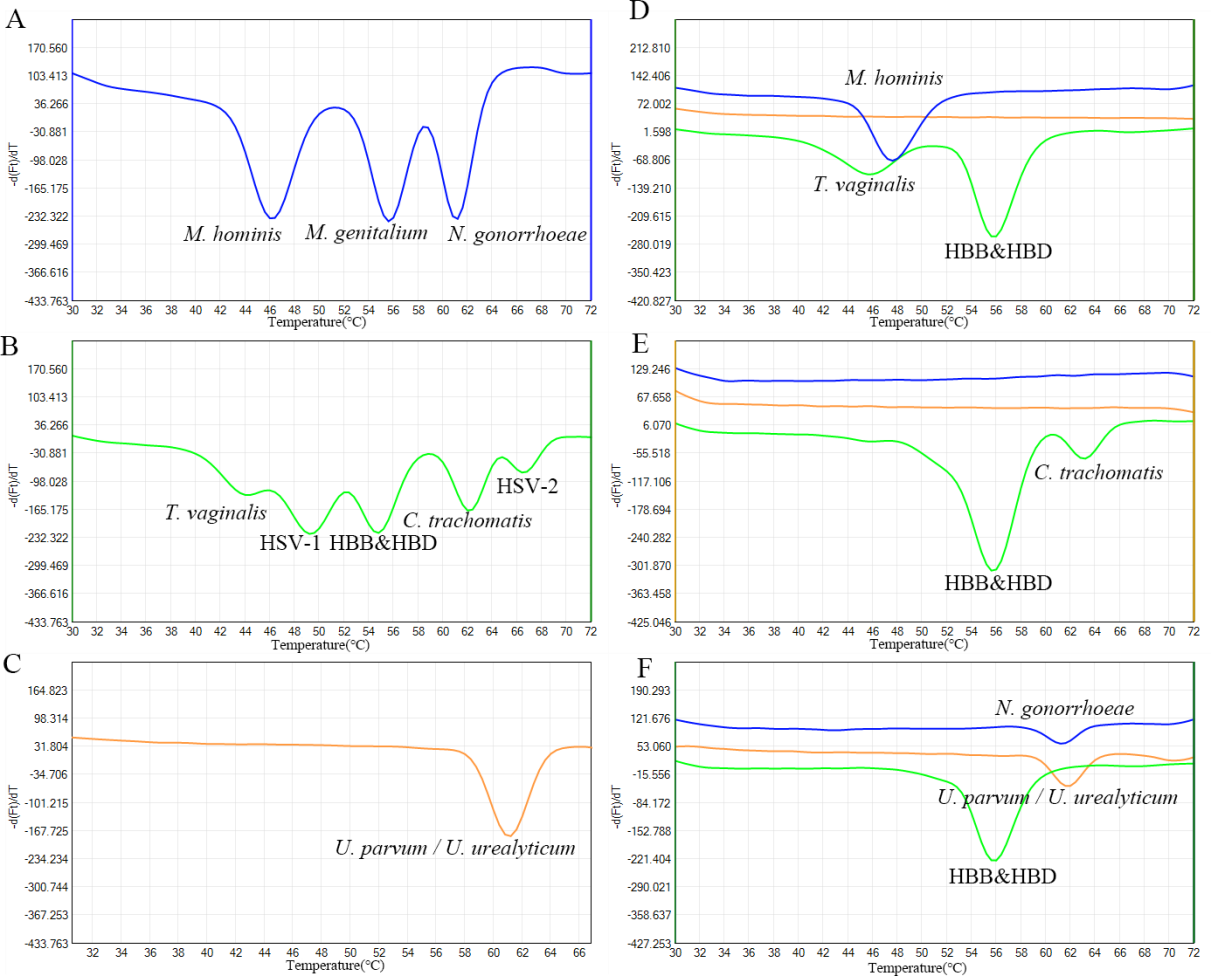
(A-C) Melting curves from mixed plasmids in FAM (A), HEX (B), and ROX (C) detection channels. (D-F) Clinical samples show co-infections with M. hominis & T. vaginalis (D), C. trachomatis (E), and N. gonorrhoeae & U. parvum/U. urealyticum (F).

In this study, we developed and tested a 2D-PCR method targeting nine different etiological agents simultaneously to identify the most common STI-related pathogens, using hemoglobin subunit beta and hemoglobin subunit delta genes (*HBB&HBD*) as an internal control. The *HBB&HBD* positive control sample was derived from DNA extracted from human whole blood. Plasmids of eight positive reference strains, each with a concentration of 10^6^ copies/μL, were mixed with human whole blood DNA according to their respective detection channels. This mixture served as the amplification template to simulate multiple infections for constructing and optimizing the methodology. As shown in Fig. 1A, the FAM channel exhibits three distinct melting peaks at different temperatures, corresponding to *M. hominis* (46°C), *M. genitalium* (55.6°C), and *N. gonorrhoeae* (61.2°C), respectively. Fig. 1B shows the HEX channel with five distinct melting peaks at different temperatures, corresponding to *T. vaginalis* (44°C), HSV-1 (49.2°C), *HBB&HBD* (54.8°C), *C. trachomatis* (62°C), and HSV-2 (66.4°C). Fig. 1C shows the ROX channel with a melting peak at 61.2°C, corresponding to *U. parvum*/*U. urealyticum*. Fig. 1D shows the melting curve obtained using the 2D-PCR method to detect a cervical brush sample co-infected with *M. hominis* and *T. vaginalis*. Fig. 1E represents a cervical brush sample positive for *C. trachomatis*, and Fig. 1F depicts a cervical brush sample co-infected with *N. gonorrhoeae* and *U. parvum*/*U. urealyticum*.

**Fig 2:**
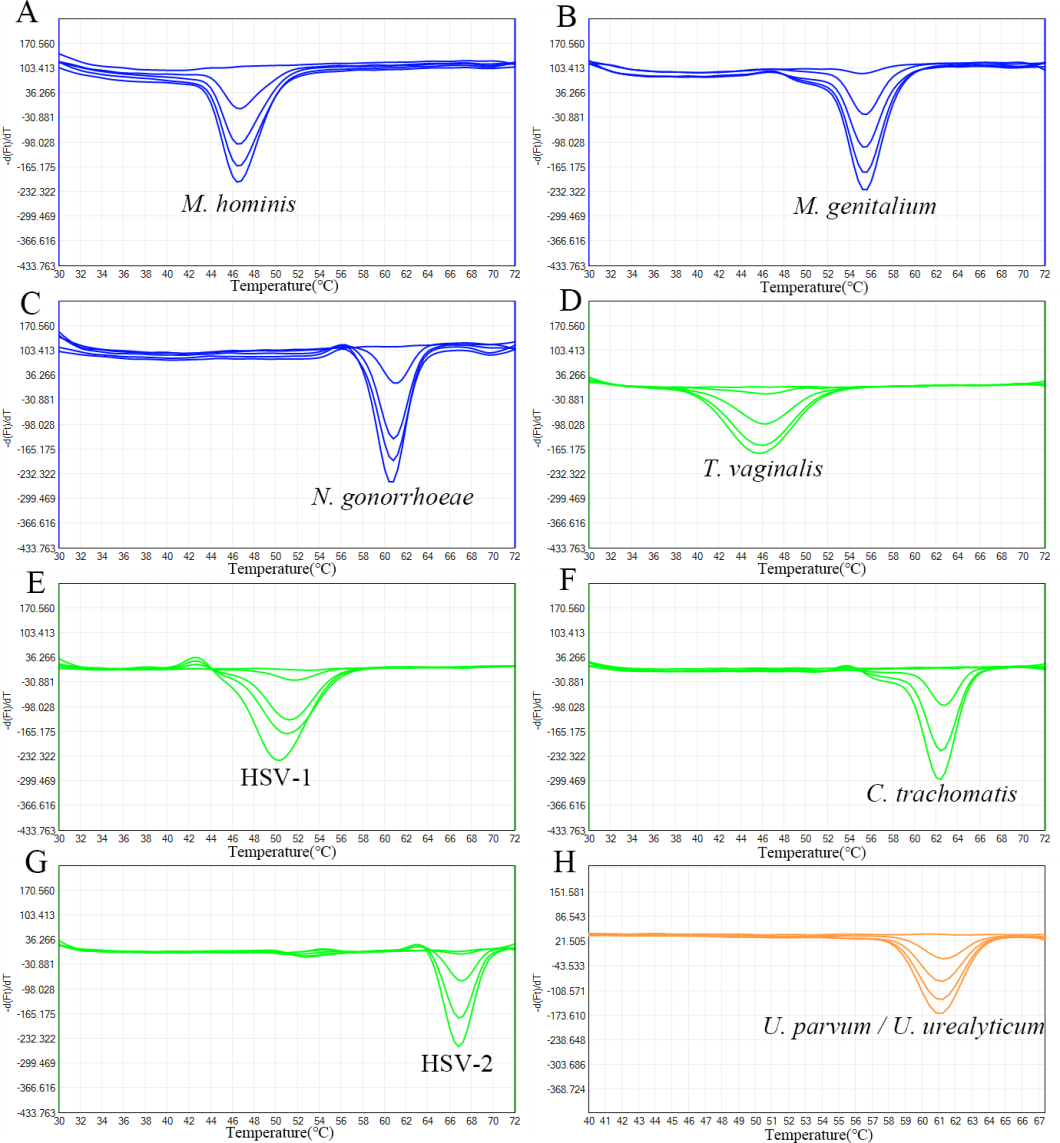
Melting curves of pathogen-specific plasmids: M. hominis (A), M. genitalium (B), N. gonorrhoeae (C), T. vaginalis (D), HSV-1 (E), C. trachomatis (F), HSV-2 (G), U. parvum / U. urealyticum (H) at serial dilutions (10⁵–10¹ copies/μL).

The analytical sensitivity of the 2D-PCR method in this study was determined by testing serial dilutions of positive standards ranging from 10^5^ to 10^1^ copies/μL of plasmids carrying the target genes of each agent. As shown in Fig. 2, with the decreasing concentration of plasmids, the depth of each melting peak gradually becomes shallower. The melting peaks of all nine STDPs can still be clearly distinguished at concentrations of either 10^2^ or 10^3^ copies/μL. Among them, the LOD for *M. hominis, M. genitalium, N. gonorrhoeae,* and *U. parvum*/*U. urealyticum* is 10^2^ copies/μL, while the LOD for *T. vaginalis*, HSV-1, *C. trachomatis*, and HSV-2 is 10^3^ copies/μL.

### Analysis of consistency in identification results between 2D-PCR and triple real-time PCR

A total of 2,193 cervical brush samples were tested using both the 2D-PCR method and the triplex real-time fluorescence quantitative PCR method. Consistency analysis was performed on the results obtained from the two methods. As shown in Table 1, the overall detection consistency between the two methods is very high, with a kappa value of 0.90. Specifically, the kappa value for *U. parvum*/*U. urealyticum* is 0.91, for *M. hominis* is 0.98, for *T. vaginalis* is 0.91, for *M. genitalium* is 0.82, for *N. gonorrhoeae* is 1, for *C. trachomatis* is 1, and for HSV-2 is 1.

**TABLE 1:** Analysis of consistency in identification results between 2D-PCR and triple real-time PCR

STDPs	2D-PCR	Triple real-time PCR		Kappa
		Positive	Negative	
*U. parvum/U. urealyticum*	Positive	696	4	0.91
	Negative	81	1,412	
*M. hominis*	Positive	165	7	0.98
	Negative	0	2,021	
*T. vaginalis*	Positive	20	4	0.91
	Negative	0	2,169	
*M. genitalium*	Positive	7	0	0.82
	Negative	3	2,183	
*N. gonorrhoeae*	Positive	2	0	1
	Negative	0	2191	
*C. trachomatis*	Positive	3	0	1
	Negative	0	2,190	
HSV-2	Positive	2	0	1
	Negative	0	2,191	
Total	Positive	755	14	0.90
	Negative	85	1,339	

### Epidemiological investigation of co-infection of STDPs and HPV

**Fig 3:**
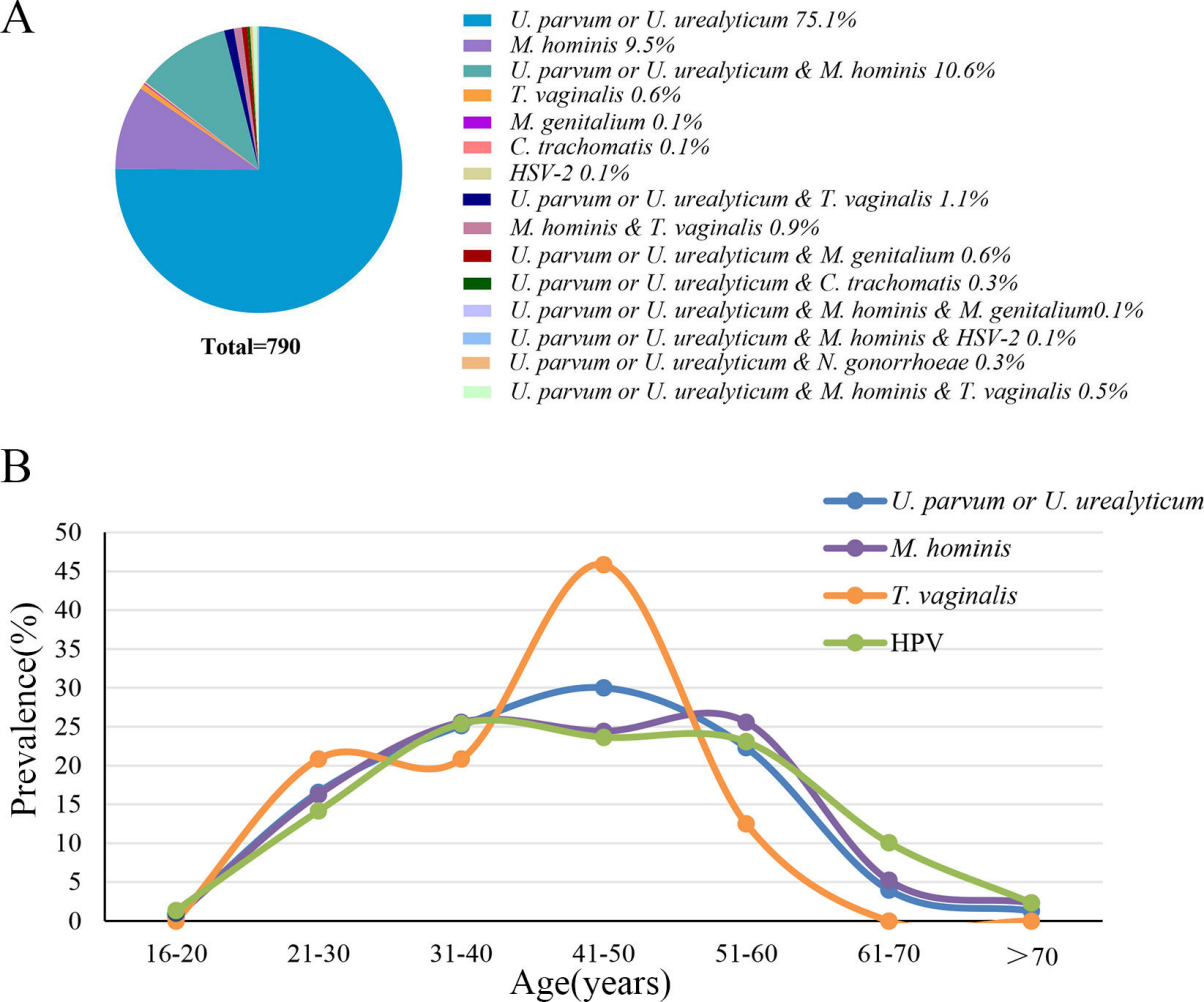
(A) Pie chart showing distribution of pathogenic microorganisms in 790 STDPs-positive patients. (B) The age distribution curves of the three most prevalent STDPs (U. parvum/U. urealyticum, M. hominis, and T. vaginalis) and HPV-positive individuals.

A statistical analysis was conducted on the STDPs and HPV infection status in 2,193 cervical brush samples. The median age was 45 years (interquartile range [IQR], 35–53). A total of 48.6% (1,066/2,193) tested positive for one or more pathogens, while 51.4% (1,127/2,193) tested negative. One or more STDPs were detected in 36.02% (790/2,193) of patients, with a median age of 44 years (IQR 33.75–51). Specifically, the proportion of individuals with a single infection of *U. parvum*/*U. urealyticum* is 27.04% (593/2,193); *M. hominis* is 3.42% (75/2,193); *T. vaginalis* is 0.23% (5/2,193); and *M. genitalium*, *C. trachomatis*, or HSV-2 is 0.05% (1/2,193). STI co-infections represented 5.20% (114/2,193) of cases: *U. parvum*/*U. urealyticum* and *M. hominis* (*n* = 84), *U. parvum*/*U. urealyticum* and *T. vaginalis* (*n* = 8), and *M. hominis* and *T. vaginalis* (*n* = 7). The distribution of pathogenic microorganisms among populations infected with STDPs (n=790) is shown in Fig. 3A.

Fig. 3B illustrates the age distribution curves of the three most prevalent STDPs (*U. parvum*/*U. urealyticum*, *M. hominis*, and *T. vaginalis*) and HPV-positive individuals. The age-stratified analysis shows that the populations infected with *U. parvum*/*U. urealyticum* and *T. vaginalis* are primarily within the age bracket of 41 to 50 years. The population infected with *M. hominis* is evenly distributed across the age ranges of 31–40 and 51–60 years. The HPV-infected population is predominantly within the age range of 31 to 40 years.

Table 2 shows the correlation between STDPs and HPV infections. Among 2,193 patients, 516 were HPV positive (23.53%), while 1,677 were HPV negative (76.47%). Among the HPV-positive patients, 239 were also infected with STDPs, accounting for 46.32% of the total number of HPV-positive cases. In the HPV-negative population, the number of individuals carrying STDPs was 551, accounting for 32.86% of the HPV-negative group. Therefore, the proportion of HPV-positive patients carrying STDPs is higher than that in the HPV-negative population. Specifically, *U. parvum*/*U. urealyticum* and *M. hominis* are associated with HPV infection. In the HPV-positive group, 39.92% carried *U. parvum*/*U. urealyticum*, and 14.1% carried *M. hominis*. In contrast, infections with *T. vaginalis* and *M. genitalium* showed no significant correlation with HPV infection.

**TABLE 2:** Correlation analysis of different STDPs and HPV co-infection

STDPs		HPV positive (*n* = 516)	HPV negative (*n* = 1677)	χ²	*P*-value
*U. parvum*/ *U. urealyticum*	Positive	206 (39.92%)	494 (29.50%)	19.89	**<0.0001^*a*^**
	Negative	310 (60.08%)	1,183 (70.50%)		
*M. hominis*	Positive	73 (14.10%)	99 (5.90%)	37.10	**<0.0001^*a*^**
	Negative	443 (85.90%)	1,578 (94.10%)		
*T. vaginalis*	Positive	7 (1.40%)	17 (1.00%)	0.43	0.5127
	Negative	509 (98.60%)	1,660 (99.00%)		
*M. genitalium*	Positive	2 (0.39%)	5 (0.30%)	0.10	0.7528
	Negative	514 (99.61%)	1,672 (99.70%)		
*N. gonorrhoeae*	Positive	2 (0.39%)	0 (0%)	–^*b*^	0.0553
	Negative	514 (99.61%)	1,677 (100%)		
*C. trachomatis*	Positive	1 (0.19%)	2 (0.12%)	–^*b*^	0.5530
	Negative	515 (99.81%)	1,675 (99.88%)		
HSV-2	Positive	1 (0.19%)	1 (0.06%)	–^*b*^	0.4153
	Negative	515 (99.81%)	1,676 (99.94%)		
STDPs	Positive	239 (46.32%)	551 (32.86%)	31.03	**<0.0001^*a*^**
	Negative	277 (53.68%)	1,126 (67.14%)		

^*a*^ *P* value less than 0.05 were bold.

^*b*^ The “(-)” indicates that Fisher’s exact test was used here, and the chi-square value cannot be provided.

At the same time, we analyzed the correlation between multiple STDP infections and multiple HPV infections. Among the 239 patients simultaneously infected with STDPs and HPV, 14 (5.85%) samples were simultaneously infected with multiple HPVs and multiple STDPs, 145 (60.67%) samples were infected with a single HPV and a single STDP, 41 samples were infected with multiple HPVs while also being infected with one STDP, and 30 samples were infected with multiple STDPs while also being infected with one HPV type. Statistical analysis indicates that there is no significant correlation between multiple STDP infections and multiple HPV infections (Table S5).

Table 3 shows the correlation between different types of STDPs and high-risk (HR) and low-risk (LR) HPV infections. Among them, the infection of *U. parvum*/*U. urealyticum* was associated with high-risk or low-risk HPV infections. Among the 516 HPV-positive cases, there were 353 cases of HR-HPV infection, 110 cases of LR-HPV infection, and 53 cases with co-infection of both high-risk and low-risk HPV (HLR-HPV). Among the HR-HPV-infected individuals, 124 cases (35.13%) were also infected with *U. parvum*/*U. urealyticum*. In the LR-HPV-infected group, 55 cases (50%) were co-infected with *U. parvum*/*U. urealyticum*. Among patients with co-infection of HLR-HPV, 27 cases (50.94%) were also infected with *U. parvum*/*U. urealyticum*. Therefore, the prevalence of *U. parvum*/*U. urealyticum* was higher in the LR-HPV group and among those with co-infection of HLR-HPV.

**TABLE 3:** Correlation analysis between infections of different STDPs and infections of high-risk and low-risk HPV types

STDPs		HR-HPV (*n* = 353)	LR-HPV (*n* = 110)	HLR-HPV (*n* = 53)	χ²	*P*-value
*U. parvum/* *U. urealyticum*	Positive	124 (35.13%)	55 (50.00%)	27 (50.94%)	10.73	**0.0047^*a*^**
	Negative	229 (64.87%)	55 (50.00%)	26 (49.06%)		
*M. hominis*	Positive	45 (12.75%)	18 (16.36%)	10 (18.87%)	1.986	0.3704
	Negative	308 (87.25%)	92 (83.64%)	43 (81.13%)		
*T. vaginalis*	Positive	4 (1.13%)	3 (2.73%)	0 (0%)	2.405	0.3004
	Negative	349 (98.87%)	107 (97.27%)	53 (100%)		
*M. genitalium*	Positive	1 (0.28%)	0 (0%)	1 (1.89%)	3.613	0.1642
	Negative	352 (99.72%)	110 (100%)	52 (98.11%)		
*N. gonorrhoeae*	Positive	1 (0.28%)	1 (0.91%)	0 (0%)	1.081	0.5826
	Negative	352 (99.72%)	109 (99.09%)	53 (100%)		
*C. trachomatis*	Positive	0 (0%)	1 (0.91%)	0 (0%)	3.698	0.1574
	Negative	353 (100%)	109 (99.09%)	53 (100%)		
HSV-2	Positive	0 (0%)	1 (0.91%)	0 (0%)	3.698	0.1574
	Negative	353 (100%)	109 (99.09%)	53 (100%)		
STDPs	Positive	147 (41.64%)	61 (55.45%)	31 (58.49%)	9.954	**0.0069^*a*^**
	Negative	206 (58.36%)	49 (44.55%)	22 (41.51%)		

^*a*^ *P* value less than 0.05 were bold.

The prevalence of genotypes in individuals with and without concomitant STDP infections is shown in Table 4. HPV 52 was the most frequent HPV type in individuals with concomitant STDPs, accounting for 6.2% (49/790), followed by HPV 61 at 4.6% (36/790) and HPV 53 at 3.9% (31/790). On the other hand, in individuals without concomitant STDP infections, HPV 52 was also the most common genotype, accounting for 3.8% (53/1,403), followed by HPV 16 at 2.4% (33/1,403) and 61 at 2.2% (31/1,403). HPV 52, 53, 6, 11, 42, 43, and 61 were significantly more prevalent in individuals with concomitant STDP infections (*P* < 0.05).

**TABLE 4:** Prevalence of HPV types in patients tested positive and negative for STDPs^*a*^

HPV types	STDPs Positive (*n* = 790)	STDPs Negative (*n* = 1403)	χ²	*P*-value
HPV16	28	33	2.656	0.1031
HPV18	8	12	0.139	0.7098
HPV31	5	6	0.115	0.7351
HPV33	3	8	0.085	0.7708
HPV35	6	5	0.937	0.3330
HPV39	11	18	0.0464	0.8295
HPV45	2	4	0.0831	0.7731
HPV51	8	18	0.315	0.5745
HPV52	49	53	6.701	**0.0096^*a*^**
HPV53	31	25	9.32	**0.0023^*a*^**
HPV56	11	16	0.264	0.6074
HPV58	25	31	1.852	0.1735
HPV59	14	24	0.011	0.9156
HPV66	8	9	0.905	0.3414
HPV68	4	6	0.005	0.9461
HPV82	1	4	0.079	0.7788
HPV6	13	7	7.353	**0.0067^*a*^**
HPV11	5	1	3.966	**0.0464^*a*^**
HPV40	2	0	–^*b*^	0.1297
HPV42	4	0	4.607	**0.0318^*a*^**
HPV43	13	7	7.353	**0.0067^*a*^**
HPV44	10	13	0.5605	0.4541
HPV55	5	6	0.1145	0.7351
HPV61	36	31	9.403	**0.0022^*a*^**
HPV81	8	5	2.664	0.1026
HPV83	2	3	0.079	0.7788

^*a*^ *P* value less than 0.05 were bold.

^*b*^ The “(-)” indicates that Fisher’s exact test was used here, and the chi-square value cannot be provided.

## DISCUSSION

According to the 2021 policy released by the Chinese government, efforts will be made to progressively increase the cervical cancer screening coverage rate, aiming for over 50% among eligible women by the end of 2025 (5). Recent expert consensus and guidelines recommend HPV genotyping as the primary screening method for cervical cancer (6). Cervical cancer development is multifactorial, infections with other STDPs can disrupt the cervical microenvironment, increasing the risk of HPV infection and potentially promoting tumorigenesis alongside HPV (7). Therefore, co-testing for other STDPs with HPV screening would enable clinicians to provide precise and effective personalized treatments.

Most STDP diagnostic test kits on the market utilize PCR methods (8). However, many of these kits either detect a limited number of STDPs or require multiple reaction tubes for multiplex detection, inadvertently increasing testing costs. Our research group has developed a 2D-PCR technology to simultaneously identify multiple target genes or SNPs in a closed tube using base-quenched probe technology and fluorescence melting temperature analysis. In this research, we developed a single-tube assay capable of detecting 9 STDPs simultaneously with the 2D-PCR method, making concurrent and cost-effective screening of HPV and STDPs feasible.

After developing, optimizing, and validating the 2D-PCR detection method, we investigated the prevalence of STDPs in 2,193 individuals who underwent HPV screening. The results were compared with triplex real-time fluorescent quantitative PCR for consistency, showing high overall consistency (kappa = 0.90). For *U. parvum*/*U. urealyticum*, the sensitivity of 2D-PCR was inferior to triplex real-time PCR, with 81 positive cases by qPCR not detected by 2D-PCR. To enhance sensitivity for *U. parvum*/*U. urealyticum*, we conducted various optimizations, including designing specific primers, matching them with different tags, and assigning *U. parvum*/*U. urealyticum* to a separate channel to prevent probe competition during amplification. The concordance for detecting *M. hominis* and *T. vaginalis* was 0.98 and 0.91, respectively, with 2D-PCR showing superior sensitivity compared with qPCR. The kappa value for *M. genitalium* was 0.82, potentially due to the limited number of positive cases.

In our study, *U. parvum*/*U. urealyticum* were the predominant STDPs, representing 31.92% of cases, with *M. hominis* next at 7.84% (including multiple infections). A study from Shanghai (2016–2021) found a similar prevalence of *U. urealyticum* (9). *U. parvum* and *U. urealyticum* are associated with various clinical manifestations, notably adverse pregnancy outcomes like chorioamnionitis and preterm premature rupture of membranes leading to preterm birth (10). Evidence suggests a causal role for *U. parvum*/*U. urealyticum* in nongonococcal urethritis and male infertility (11). In Xi’an, China, the prevalence of *M. hominis* was 6.48% (12), similar to our results. *M. hominis*, as an endosymbiont of *T. vaginalis*, typically co-infects (13). In our study, of 24 *T. vaginalis-*positive cases, 11 were also positive for *M. hominis. M. hominis* is linked to various diseases, including pelvic inflammatory disease, cervicitis, and pyelonephritis (14). Genital herpes, a chronic sexually transmitted infection caused by HSV-1 or HSV-2, is characterized by recurrent genital ulcers. While 2D-PCR is highly sensitive and specific, false-negative results can occur. For instance, swabs taken without genital ulcers may lack sensitivity due to intermittent genital HSV shedding (15). In our study, HSV-1 was not detected, while HSV-2 was found in only two cases. Another reason may be that patients with genital herpes symptoms often seek care at STD clinics rather than gynecological clinics.

Our study found higher infection rates of *U. parvum*/*U. urealyticum* and *M. hominis* in the HPV-positive population, consistent with previous findings (16, 17). Research indicates that these infections may increase HPV risk by affecting immune response balance or due to lifestyle factors, leading to higher HPV and STDP rates (18). *U. parvum* and *U. urealyticum* are also linked to HPV persistence and early cervical cytological changes (19). Therefore, treating these infections during HPV treatment may help prevent early cervical cancer progression. Our data showed no correlation between multiple STDP coinfections and multiple HPV genotypes, but *U. parvum*/*U. urealyticum* infection rates were higher in low-risk HPV populations, a novel finding requiring further investigation. We analyzed specific HPV genotypes and STDP infections, finding associations with HPV types 52, 53, 6, 11, 42, 43, and 61. Types 52 and 53 are HR-HPV, while the others are LR-HPV, which are less associated with cervical cancer than types 16 or 18, raising questions about whether STDP co-infections accelerate cervical lesion progression.

In fact, we also developed a 2D-PCR method for the identification and genotyping of 16 HR-HPV types and related tumor suppressor genes p53 and RB1 for cervical cancer (20). Therefore, using 2D-PCR as a cost-effective screening method that can simultaneously detect HPV and other STDPs would have significant clinical and economic value. This approach enables early detection, prevention, and management of these infections, leading to reduced disease burden and healthcare costs.

## MATERIALS AND METHODS

### Study population and clinical specimens

This study included 2,193 women who underwent routine gynecological inspections from November 2022 to March 2023 at the Third Affiliated Hospital of Soochow University. Inclusion criteria: women of reproductive age over 18 years old; having sexual experience; having regular menstruation; not using any medications within 1 week; and no vaginal douching, cervical treatment, or sexual intercourse within 72 h. Exclusion criteria: women who are pregnant or lactating and women with chronic diseases requiring long-term medication. Exfoliated cervical cells were obtained from the ecto- and endo-cervix portions of the uterus using a cytobrush. With approval from the Institutional Ethics Committee (approval number: [2022 (ke) No. 046]), residual cervical swab samples that could not be identified individually were used.

### HPV DNA detection and genotyping

HPV testing was conducted using the Tellgenplex HPV 27 genotyping assay (Tellgen Corporation, Shanghai, China) on the Luminex 200 platform (Luminex Corporation, Austin, TX). The assay is a flow cytometry fluorescence hybridization method that detects 17 high-risk HPV types (HPV16, 18, 26, 31, 33, 35, 39, 45, 51, 52, 53, 56, 58, 59, 66, 68, and 82) and 10 low-risk HPV types (HPV 6, 11, 40, 42, 43, 44, 55, 61, 81, and 83).

### STDP detection

The residual DNA from HPV genotyping was utilized for the detection of STDPs.

#### Preparation of positive control plasmids

Plasmids containing the highly conserved sequence regions of the nine STDPs were synthesized by Sangon Biotech Co., Ltd. (Shanghai, China). The pUC57 vector containing the target fragments was cloned and amplified in *E. coli* JM109 cells, followed by extraction and purification.

#### 2D-PCR primers and probes

Primers for the nine STDPs were designed using the software Primer Premier 5.0 (Premier Biosoft Intl., California, USA) and tested with NCBI BLAST to ensure specificity. According to the principles of 2D-PCR, a tag homologous to the probe was linked to the 5′ end of the forward primer, with several mismatched bases between the probe sequence and the complementary sequence of the tag. Only one probe is required for each detection channel. Three probes required for the detection of the nine STDPs were labeled with carboxyfluorescein (FAM), hexachloro-fuorescein (HEX), and Alexa 568, respectively. Both primers and probes were synthesized by Sangon Biotech Co., Ltd. (Shanghai, China). The primers and probes are listed in Table S1.

#### 2D-PCR reaction

The formulation of the 2D-PCR reaction system is shown in Table S2. PCR amplifications and melting curve analyses were performed using a SLAN-96S real-time PCR machine (Hongshi Tech, Shanghai, China). Cycling conditions included preincubation at 95°C for 10 min, followed by amplification for a total of 40 cycles under the following conditions: denaturation at 95°C for 5 s and annealing at 60°C for 15 s. The fluorescence acquisition began with heating at 95°C for 30 s and then at 30°C for 4 min; the temperature was gradually increased from 30 to 72°C with a ramp rate of 0.06°C/s, during which the fluorescence signal was acquired continuously. Fluorescence intensity was measured using three detection channels: FAM, HEX, and ROX. Plasmids containing nine STDPs with a concentration of 10^6^ copies/μL and human whole blood DNA were thoroughly mixed according to their detection channels to simulate multiple infections as the amplification template for methodological optimization. The assay targets *HBB* and *HBD* as an internal control to monitor DNA purification efficiency, PCR inhibition, and cell adequacy.

#### Triple RT-PCR primers and probes

Primers and probes for nine STDPs were designed using Primer Premier 5.0 (Premier Biosoft) and validated for specificity using NCBI BLAST. In each PCR reaction, three probes were labeled with FAM, ROX, and VIC fluorescent dyes, respectively. The primers and probes are listed in Table S3. The formulation of the reaction system is shown in Table S4. Amplification began with an initial denaturation step at 95°C for 10 min, followed by 40 cycles of denaturation at 95°C for 10 s and annealing/extension at 60°C for 15 s. Fluorescence acquisition started with a 4 min incubation at 30°C, followed by a gradual temperature increase from 30°C to 80°C at a ramp rate of 0.1°C/s, during which the fluorescence signal was continuously monitored. The final step involved cooling at 40°C for 30 s.

#### Sensitivity experiments of the 2D-PCR detection system

To evaluate the sensitivity of the 2D-PCR detection system constructed in this study, the STD positive control plasmids were diluted with TE buffer to concentrations of 10^5^, 10^4^, 10^3^, 10^2^, and 10^1^ copies/μL. Then, 5 µL of the positive control plasmids was subjected to the 2D-PCR assay to analyze the lowest template concentration detectable by this method.

### Statistical analysis

Categorical variables were represented as proportions, while median and interquartile range (IQR) values were calculated for continuous variables. The consistency of the two detection methods was assessed using the SPSSAU online analysis software by calculating the kappa value. The chi-squared test (*χ*^*2*^) was used to compare categorical variables across groups. The concordance rates were analyzed using the kappa test, with kappa values of 0.2, 0.2–0.4, 0.4–0.6, and >0.6 considered as poor, fair, moderate, and good agreement, respectively.

## Supplementary Material

Reviewer comments

## Data Availability

Upon submission, authors agree to make any materials, data, and associated protocols available upon request.

## SUPPLEMENTAL MATERIAL

Supplemental material spectrum.00237-25-s0001.pdf Tables S1 to S5.

OPEN PEER REVIEW reviewer-comments.pdf An accounting of the reviewer comments and feedback.

ASM does not own the copyrights to Supplemental Material that may be linked to, or accessed through, an article. The authors have granted ASM a non-exclusive, world-wide license to publish the Supplemental Material files. Please contact the corresponding author directly for reuse.

## References

[1] WHO. 2023. The diagnostics landscape for sexually transmitted infections. World Health Organization, Geneva.

[2] Li K, Li Q, Song L, Wang D, Yin R. 2019. The distribution and prevalence of human papillomavirus in women in mainland China. Cancer 125:1030–1037. doi:10.1002/cncr.32003 30748006

[3] Yuan Y, Cai X, Shen F, Ma F. 2021. HPV post-infection microenvironment and cervical cancer. Cancer Lett 497:243–254. doi:10.1016/j.canlet.2020.10.034 33122098

[4] Usyk M, Zolnik CP, Castle PE, Porras C, Herrero R, Gradissimo A, Gonzalez P, Safaeian M, Schiffman M, Burk RD, Costa Rica HPV Vaccine Trial (CVT) Group. 2020. Cervicovaginal microbiome and natural history of HPV in a longitudinal study. PLoS Pathog 16:e1008376. doi:10.1371/journal.ppat.1008376 32214382 PMC7098574

[5] Qiu L, Chen F, Zhao W, Meng Y, Wang Y, Cheng W, Yang Q, Sui L, Wei L, Di W. 2024. 2024 cervical cancer screening and early precision diagnosis status white paper. Chin J Pract Gynecol Obstet 40:85–95. doi:10.19538/j.fk2024010118

[6] WHO. 2024. WHO guideline for screening and treatment of cervical pre-cancer lesions for cervical cancer prevention: use of dual-stain cytology to triage women after a positive test for human papillomavirus (HPV). World Health Organization, Geneva. 38976622

[7] Huang R, Liu Z, Sun T, Zhu L. 2024. Cervicovaginal microbiome, high-risk HPV infection and cervical cancer: mechanisms and therapeutic potential. Microbiol Res 287:127857. doi:10.1016/j.micres.2024.127857 39121703

[8] Tuddenham S, Hamill MM, Ghanem KG. 2022. Diagnosis and treatment of sexually transmitted infections: a review. JAMA 327:161–172. doi:10.1001/jama.2021.23487 35015033

[9] Wang S, Ding L, Liu Y, Sun Z, Jiang W, Miao Y, Wang S, Meng J, Zhao H. 2023. Characteristics of common pathogens of urogenital tract among outpatients in Shanghai, China from 2016 to 2021. Front Public Health 11:1228048. doi:10.3389/fpubh.2023.1228048 38089034 PMC10711282

[10] Noda-Nicolau NM, Tantengco OAG, Polettini J, Silva MC, Bento GFC, Cursino GC, Marconi C, Lamont RF, Taylor BD, Silva MG, Jupiter D, Menon R. 2022. Genital mycoplasmas and biomarkers of inflammation and their association with spontaneous preterm birth and preterm prelabor rupture of membranes: a systematic review and meta-analysis. Front Microbiol 13:859732. doi:10.3389/fmicb.2022.859732 35432251 PMC9006060

[11] Beeton ML, Payne MS, Jones L. 2019. The role of Ureaplasma spp. in the development of nongonococcal urethritis and infertility among men. Clin Microbiol Rev 32:e00137-18. doi:10.1128/CMR.00137-18 31270127 PMC6750135

[12] Zeng XY, Xin N, Tong XN, Wang JY, Liu ZW. 2016. Prevalence and antibiotic susceptibility of Ureaplasma urealyticum and Mycoplasma hominis in Xi’an, China. Eur J Clin Microbiol Infect Dis 35:1941–1947. doi:10.1007/s10096-016-2745-2 27530531

[13] Margarita V, Congiargiu A, Diaz N, Fiori PL, Rappelli P. 2023. Mycoplasma hominis and Candidatus Mycoplasma girerdii in Trichomonas vaginalis: peaceful cohabitants or contentious roommates? Pathogens 12:1083. doi:10.3390/pathogens12091083 37764891 PMC10535475

[14] Yagur Y, Weitzner O, Barchilon Tiosano L, Paitan Y, Katzir M, Schonman R, Klein Z, Miller N. 2021. Characteristics of pelvic inflammatory disease caused by sexually transmitted disease – an epidemiologic study. J Gynecol Obstet Hum Reprod 50:102176. doi:10.1016/j.jogoh.2021.102176 34087450

[15] Johnston C. 2022. Diagnosis and management of genital herpes: key questions and review of the evidence for the 2021 centers for disease control and prevention sexually transmitted infections treatment guidelines. Clin Infect Dis 74:S134–S143. doi:10.1093/cid/ciab1056 35416970

[16] Ye H, Song T, Zeng X, Li L, Hou M, Xi M. 2018. Association between genital mycoplasmas infection and human papillomavirus infection, abnormal cervical cytopathology, and cervical cancer: a systematic review and meta-analysis. Arch Gynecol Obstet 297:1377–1387. doi:10.1007/s00404-018-4733-5 29520664

[17] Klein C, Samwel K, Kahesa C, Mwaiselage J, West JT, Wood C, Angeletti PC. 2020. Mycoplasma co-infection is associated with cervical cancer risk. Cancers (Basel) 12:1093. doi:10.3390/cancers12051093 32353967 PMC7281224

[18] Liang Y, Chen M, Qin L, Wan B, Wang H. 2019. A meta-analysis of the relationship between vaginal microecology, human papillomavirus infection and cervical intraepithelial neoplasia. Infect Agent Cancer 14:29. doi:10.1186/s13027-019-0243-8 31673281 PMC6815368

[19] Xie L, Li Q, Dong X, Kong Q, Duan Y, Chen X, Li X, Hong M, Liu T. 2021. Investigation of the association between ten pathogens causing sexually transmitted diseases and high-risk human papilloma virus infection in Shanghai. Mol Clin Oncol 15:132. doi:10.3892/mco.2021.2294 34055347 PMC8138855

[20] Zhang J, Yao S, Yu Y, Yu M, Luo G. 2024. Development of a typing detection method for high-risk human papillomavirus and related tumor suppressor genes p53 and RB1 based on two-dimensional PCR technology. Chin J Lab Med 47:391–400. doi:10.3760/cma.j.cn114452-20240204-00069

