# Supplementary material for: Establishment of a two-dimensional PCR method for simultaneous detection of nine sexually transmitted disease pathogens: insights into coinfection rates and epidemiological trends in HPV screening
Source: Microbiol Spectr. 2025 Apr 24;13(6):e00237-25. doi: 10.1128/spectrum.00237-25 (PMC12131753; doi:10.1128/spectrum.00237-25)
Supplement: Supplemental material — Tables S1 to S5. [file spectrum.00237-25-s0001.pdf]

Supplemental Table 1. Primer and probe sequences for 9 STDPs and HBB&HBD using the 2D-PCR method.

STDPs	Prime name	Primer sequence (5′→3′)[a]	Tm[b]	Probe sequence (5'→3')
M. hominis	F1	ccatctacactcagtccactgctcgaccttccttatctcTAATGACCCTGAATTTGAAATCGT	46°C	FAM-CCATCTACACTCCCAAACTAATCTTTTCTTCCTTATCTC-P
	R1	GTGAGCTAATGTAGCWGCATCTGTT		
M. genitalium	F2	ccatctacactcccatcagttcactttcttccttatctcCAACCAAAGAAAAGACTGGCTAAG	55.6°C	
	R2	AGAGAAAATAACCACCTACACCTGTT		
N. gonorrhoeae	F3	ccatctacactcccaaatcgtacttttcttccttatctcCGGCATAATACACATCCGACC	61.2°C	
	R3	ACGCCTGCTACTTTCACGCT		
T. vaginalis	F4	cctaatcatcgttcacttaccatatagctcgacgtccatAATGGATGTCTTGGCTCCTCAC	44°C	HEX-CCTAATCATCAACCACTTACCATCACTTCACCTATCCAT-P
	R4	ATGTAGTACTGTCACACCCATGCTT		
HSV-1	F5	cctaatcatatcccacttaccatcttactgtgaatccatGCGTCTGTGGTGTTTTTGGC	49.2°C	
	R5	GTCTGGAGTTGGGGTTGGGT		
HBB&HBD	F6	cctaatcatcaaccacaattttaagattcacctatccatAGGTTCTTTGAGTCCTTTGGGG	54.8°C	
	R6	GAGGTTGTCCAGGTGAGCCAG		
C. trachomatis	F7	cctaatcatcaaccacgacagatcacttcacctatccatAAACTCCGCTTCCTTCAACTTAG	62°C	
	R7	CACCTACGCTCCAAGCAAAG		
HSV-2	F8	cctaatcatcaaccacacgccatcacttcacctatccatCGTCGTCTGCGCCAAATAC	66.4°C	
	R8	TCCAGGCTCGGCTGAATGT		
U. parvum / U.urealyticum	F9	cacctatccttctatcattgatttccattcaatactcctTTCCTGTTGCCCCTCAGTCT	61.2°C	Alexa fluor 568-CACCTATCCTTCTATCATTCCTTTCCATTCAATACTCCT-P
	R9	AAGGTCAAGGTATGGAAGATCCAA		

[a] The tag sequences, which are homologous to the probe sequences, are indicate in lowercase letters.

[b] Tm refers to the melting temperature of the tag sequence in the amplification product.

Supplemental Table 2. Composition of the 2D-PCR reaction system.

Component	Concentration	Volume(μL)	Final concentration
10 × Immo Buffer	10×	2.5	1×
$MgCl_2$	50 mM	0.5	1mM
dNTPs	4×2.5 mM	0.7	0.28 mM
Immolase DNA Polymerase	5U/μL	0.5	0.1 U/μL
P-FAM	10 μM	0.6	0.24 μM
P-HEX	10 μM	0.6	0.24 μM
P- Alexa fluor 568	10 μM	0.6	0.24 μM
F3/F5	10 μM	0.1	0.04 μM
F1/F2/F4/F6/F8/F9	10 μM	0.15	0.06 μM
F7	10 μM	0.2	0.08 μM
R1~R9	10 μM	0.6	0.24 μM
DNA Template	-	5	-
Purified Water	-	7.3	-
Total	-	25	-

Supplemental Table 3. Primer and probe sequences for 9 STDPs using the triplex real-time PCR.

Primer and probe name	Primer sequence (5′→3′)
F-*U. parvum / U. urealyticum*	TTCCTGTTGCCCCTCAGTCT
R-*U. parvum / U. urealyticum*	AAGGTCAAGGTATGGAAGATCCAA
P- *U. urealyticum*	ROX-ACCACAAGCACCTGCTACGATTTGTTC-BHQ2
P-*U. parvum*	6-FAM-TCCACAAGCTCCAGCAGCAATTTG-BHQ1
F-*C. trachomatis*	AAACTCCGCTTCCTTCAACTTAG
R-*C. trachomatis*	CACCTACGCTCCAAGCAAAG
P-*C. trachomatis*	VIC-TTGAATCAAGCTGTGGTTGAGCTTTATACAGA-BHQ1
F-*N. gonorrhoeae*	CGGCAGCATTCAATTTGTT
R- *N. gonorrhoeae*	AAAAAGCCGCCATTTTTGTA
P- *N. gonorrhoeae*	6-FAM-AAAACAGCAAGTCCGCCTATACGCCT-BHQ1
F-*T. vaginalis*	CGGTCGAGAAGCATGGGTG
R- *T. vaginalis*	TCAGTTCAGCGGGTCTTCC
P- *T. vaginalis*	VIC-ATAAGACAAACAACACGTAGTCTGCCATACG-BHQ1
F-*M. genitalium*	CAACCAAAGAAAAGACTGGCTAAGA
R-*M. genitalium*	AGAGAAAATAACCACCTACACCTGTT
P-*M. genitalium*	ROX-TCTTGAGCCTTTCTAACCGCTGCACTT-BHQ2
F-HSV-1	CTGTGGTGTTTTTGGCATCA
R-HSV-1	GGTGGTGGAGGAGACGTTG
P-HSV-1	6-FAM-TTATCCCATTCCTTTTGGTTCTTGTCGG-BHQ1
F-*M. hominis*	GGTTTTGGYAGAATTGGTCGTYT
R-*M. hominis*	TCAWTTTTCCATGAGCTGTATCG
P-*M. hominis*	VIC-TTTTAATGACCCTGAATTTGAAATCGTTGCT-BHQ1
F-HSV-2	CAAATACGCCTTAGCAGACC
R-HSV-2	TGAATGTGGTAAACACGCTTC
P-HSV-2	ROX-CTTAAGATGGCCGATCCCAATCGATT-BHQ2

Supplemental Table 4. Composition of the triplex real-time PCR reaction system.

Component	Concentration	Volume(μL)
10 × PCR Buffer	10×	2.5
$MgCl_2$	50 mM	2.5
dNTPs	4×2.5 mM	0.5
Taq polymerase	5U/μL	0.25
Forward primer	100 μM	0.04
Reverse primer	100μM	0.04
Probe	100 μM	0.04
DNA Template	-	5
Purified Water	-	11.89
Total	-	25

Supplemental Table 5. Correlation analysis of multiple STDPs infections and multiple HPV infections.

STDPs	HPV single-type infection	HPV multi-type infection	χ^2	*P*-value
Infection with a single STDP	145	41	0.4450	0.5047
Co-infection with multiple STDPs	39	14		
